# Tissue Profile of CDK4 and STAT3 as Possible Innovative Therapeutic Targets in Urinary Bladder Cancer

**DOI:** 10.31557/APJCP.2020.21.2.547

**Published:** 2020

**Authors:** Tarek Aboushousha, Olfat Hammam, Ahmed Aref, Amira Kamel, Mohamed Badawy, Amr Abdel Hamid

**Affiliations:** 1 *Department of Pathology, *; 3 *Department of Urology, Theodor Bilharz Research Institute, Cairo, *; 2 *Faculty of Biotechnology, October University for Modern Sciences and Arts, Giza, Egypt. *

**Keywords:** CDK4-STAT3-Therapeutic targets-bladder cancer

## Abstract

Bladder cancer represents a global health problem. It ranks ninth in worldwide cancer incidence. In Egypt, carcinoma of the bladder is the most prevalent cancer, Bladder cancer has the highest recurrence rate of any malignancy. Certainly, suitable molecular diagnostic markers are required to improve the early detection of bladder cancer and then to prolong survival of patients. The present study was aimed to explore the expression of CDk4 and STAT3 in bladder cancer tissues as prospective for target therapy. Our studied groups showed higher values of CDK4 and STAT3 expression in malignant tissues (SCC andUC collectively) compared to cystitis, however, significantly higher values of CDK4 and STAT3 expression were detected in UC group compared to SCC group. Urothelial carcinomas with papillary patterns showed lower parameters of CDK4 and STAT3 expression compared to the non-papillary variant, with significant differences. Higher grades of UC showed significantly higher parameters of CDK4 and STAT3 expression compared to low grade ones. Muscle invasion increases the level of CDK4 and STAT3 expression parameters, compared to non-muscle invasive UC.

**Conclusion:**

Our results showed a good correlation of the expression patterns of both the cell cycle (CDK4) and inflammatory (STAT3) markers studied and might be helpful for suggesting more selective agents in the therapeutic scenario of bladder cancer in the near future. Potential biomarkers such as CDK4 andSTAT3 may be targets for molecular based therapeutic strategies in the prevention or management of bladder cancer. Future studies should explore molecular mechanisms of these proteins to define their roles in tumorigenesis.

## Introduction

Bladder cancer represents the fifth most common malignancy worldwide and a major cause of cancer-related morbidity and death. Incidence and mortality rates have remained relatively constant over the past four decades (Siegel et al., 2013). Urothelial carcinoma is well known for its divergent differentiation resulting in distinct, morphological variants (Amin et al., 2009), Squamous differentiation, defined by the presence of intercellular bridges, keratinization, or both, is the most common variant, occurring in up to 20% of urothelial carcinomas of the bladder, followed by glandular differentiation (Wasco, 2007). Although urothelial carcinoma with squamous differentiation may be associated with poor prognosis, conflicting data have been reported regarding the role of squamous differentiation in unfavorable clinical outcomes (Domanowska et al., 2007). Because it is not uncommon for squamous differentiation to concurrently occur with other histological variants of urothelial carcinoma, such as micropapillary, glandular, and sarcomatoid differentiation, studies that report on mixed urothelial carcinoma variants may contribute to the discrepancies (Kim et al., 2012).

The cell cycle is regulated in part by cyclins and their associated serine/threonine cyclin-dependent kinases, or CDKs. CDK4, in conjunction with the D-type cyclins, mediates progression through the G1 phase when the cell prepares to initiate DNA synthesis, this gene plays a key role in development of cancer and tumorigenesis (Stacey and Premkumar, 2013).

Signal transducer and activator of transcription 3 (STAT3) plays a prominent role in the growth and invasion of several types of solid tumors, it has a prognostic significance of the, upper urinary tract urothelial carcinoma (UTUC) and has role in the tumerogenesis (Matsuzaki , 2018).

## Materials and Methods

Our study consists of 68 urinary bladder biopsy specimens. We got tissue sections from their archival material kept in the pathology department of Theodor Bilharz Research Institute (TBRI), Cairo, Egypt. The patients came to TBRI hospital seeking medical advice for their urinary symptoms and were cystoscopically examined, biopsied and their specimens were sent for histopathological diagnosis. The cases under study consist of chronic cystitis (7 cases), squamous cell carcinoma (14 cases) and urothelial carcinoma (47 cases).

*Immunohistochemical Method*

Anti- STAT3 (F-2) antibody (Santa Cruz, clone EP1sc 8019, Lot# E0217, mouse monoclonal IgG) and Anti- Cdk4 (DCS-35) antibody (Santa Cruz, Lot# D1217, mouse monoclonal IgG) were used for immunohistochemical (IHC) detection of the STAT3 and Cdk4 in tissue. Tissue sections were processed for IHC analysis as follows. IHC examinations were carried out on 4 μm thick sections. Antigen retrieval was performed with 10 mM sodium citrate buffer, pH 6.0, at 90°C for 30 min. Sections were incubated in 0.03% hydrogen peroxide for 10 min at room temperature, to remove endogenous peroxidase activity, and then in blocking serum (0.04% bovine serum albumin, A2153, Sigma-Aldrich, Shanghai, China, and 0.5% normal goat serum X0907, Dako Corporation, Carpinteria, CA, USA, in PBS) for 30 min at room temperature. 

Antibodies were used at a dilution of 1:100, added to tissue sections and incubated overnight at 4°C. Sections were then washed three times for 5 min in PBS. Non-specific staining was blocked 5% normal serum for 30 min at room temperature. Finally, staining was developed with diaminobenzidine substrate (DAB) and sections were counterstained with hematoxylin. PBS replaced the antibody in negative controls.

All sections were examined by two pathologists, using light microscope [Scope A1, Axio, Zeiss, Germany]. Photomicrographs were taken using an attached microscope-camera [AxioCam, MRc5, Zeiss, Germany].

All procedures were done at the pathology department of Theodor Bilharz Research Institute, Cairo, Egypt.

*Quantification of protein expression*

The expression of both CDK4 and STAT3 markers was semiquantitatively estimated as the percentage and intensity of staining. The proportion reflected the fraction of positive staining cells from 0% to 100% and the intensity score represented the staining intensity (score 0: no staining, score 1: weak positive, score 2: moderate positive, score 3: strong positive). Finally, multiplication of the score of intensity by the percentage of positive cells yields a total expression score ranging from 0 to 300.

*Statistical analysis*

SPSS for Windows, version 20 was used for statistical analysis (IBM Corporation, Armonk, New York, USA). The comparisons of quantitative variables were performed between two groups using ANOVA and student t-tests. Associations between each antigen expressions and other studied variables were evaluated by Chi square test and fisher test. Correlations between variables were studied using Spearmann’s correlation test. The P value < 0.05 was considered statistically significant.

## Results

Our study consists of 68 urinary bladder biopsy specimens. We got tissue sections from their archival material kept in the pathology department of Theodor Bilharz Research Institute (TBRI), Cairo, Egypt. These patients came to TBRI hospital seeking medical advice for their urinary symptoms and were cystoscopically examined and biopsied for histopathological diagnosis.

The cases under-study consists of chronic cystitis (7 cases), squamous cell carcinoma (14 cases) and urothelial carcinoma (47 cases).

All cases of squamous cell carcinoma showed high tumor grade and positive muscle invasion, while most cases of urothelial carcinoma were of low grade malignancy (65.2%) and showed negative muscle invasion (69,6%). The difference was statistically significant (p<0.001).

Urothelial carcinoma showed significantly higher percent and score of CDK4 expression compared to both cases of cystitis and squamous cell carcinoma (p<0.05). Also, cases of urothelial carcinoma showed highly significant elevated STAT3 percent and scores of expression compared to cases of cystitis and squamous cell carcinoma (p<0.01). On the contrary, no significant differences in both CDK4 and STAT3 expression parameters were achieved between cases of cystitis and squamous cell carcinoma (p>0.05) (Table 1 and Histogram 1).

The mean score of STAT3 expression was significantly higher in high grade urothelial carcinoma compared to low grade one (p<0.05). No significant differences were detected in percent of cellular expression of both CDK4 and STAT3 between high and low grade urothelial carcinoma cases (p>0.05) (Table 2).

Non-papillary UC showed higher percent and scores of *STAT3* expression compared to papillary UC. The differences were highly significant (p<0.01). On the contrary, no significant differences were detected in both the percent and the score of *CDK4* expression between non-papillary and papillary UC cases (p>0.05) (Table 2).

As regards muscle invasion, cases of muscle invasive bladder cancer (MIBC) showed significantly higher score of *STAT3* expression compared to cases of non muscle invasive bladder cancer (NMIBC) (p<0.05).

No significant differences were detected in *CDK4 *expression parameters or *STAT3* percent were detected between both NMIBC AND MIBC groups (p>0.05) (Table 2).

Regarding the immunohistochemical study of urothelial carcinoma cases, there were no significant differences in both *CDK4* and *STAT3* expression parameters between bilharzial associated and non-associated groups (p>0.05) (Table 2).

**Table 1:** Differences in CDK4 and STAT3 Percentage and Scores of Expression between Studied Groups

Diagnosis		CDK4 percent	CDK4 score	STAT3 percent	STAT3 score
Cystitis (7)	Mean	0	0	2.14	2.14
	SEM	0	0	1.49	1.49
Uc (47)	Mean	18.72*	20.00*	39.46**	87.87**
	SEM	3.86	3.9	4.68	13.68
Scc (14)	Mean	4.28^	4.28^	12.14^^	20.00^^
	SEM	2.21	2.21	3.3	5.34

*, Significant difference with cases of cystitis (p<0.05); **, High significant difference with cases of cystitis (p<0.01); ^, Significant difference with cases of UC (p<0.05); ^^, High significant difference with cases of UC (p<0.01).

**Histogram 1:**
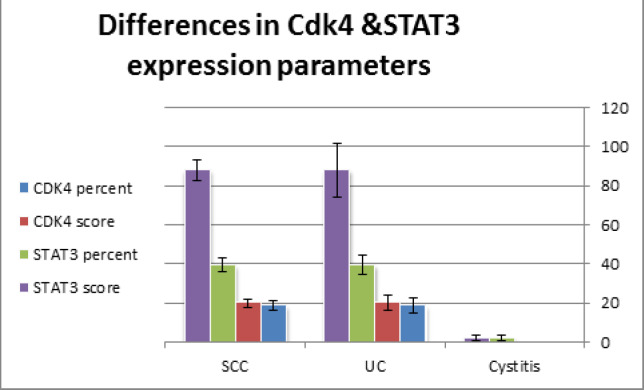
Differences in *Cdk4* and *STAT3* Expression Parameters

**Table 2:** Comparison between Expression Parameters of CDK4 and STAT3 in Subgroups of Urothelial Carcinoma (UC) as Regards to Tumor Grade, Papillary Pattern, Muscle Invasion and Bilharzial Association

Papillary pattern					
Negative	Mean	25	25	66.87	163.12
	N	16	16	16	16
	SEM	8.8	8.8	6.63	24.32
positive	Mean	15.48	17.41	25.32**	49.03**
	N	31	31	31	31
	SEM	3.73	3.87	4.48	11.61
Grade( Low / High)		CDK4 percent	CDK4 score	STAT3 percent	STAT3 score
high	Mean	12	12	40.33	96.66
	N	16	16	16	16
	SEM	4.82	4.82	6.54	19.25
low	Mean	18.7	20.64	26.29	48.70*
	N	31	31	31	31
	SEM	3.95	4.03	4.3	10.48
Muscle invasion					
NMIBC	Mean	19.09	20.9	27.72	50.3
	N	33	33	33	33
	SEM	3.87	3.94	4.16	9.96
MIBC	Mean	11.07	11.07	39.64	98.21 *
	N	14	14	14	14
	SEM	4.96	4.96	7	20.57
Bilharzial association					
Negative	Mean	14.21	15.26	32.36	71.05
	N	41	41	41	41
	SEM	3.8	3.87	5.05	14.23
positive	Mean	11.81	11.81	17.72	34.09
	N	6	6	6	6
	SEM	7.87	7.87	7.09	14.57

*, Significant difference between comparable groups (p<0.05); **, High Significant difference between comparable groups (p<0.01)

**Histogram 2:**
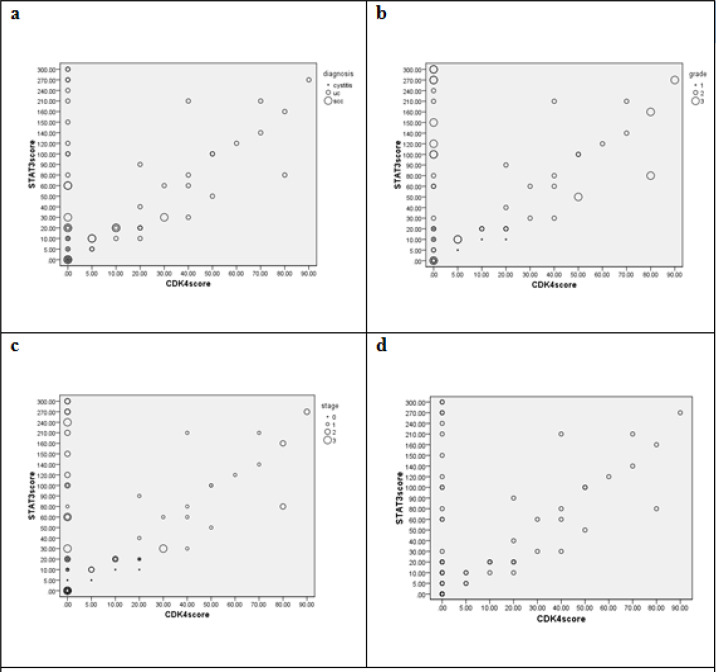
Correlations between CDK4 and STAT3 Expression Scores in View of Studied Groups (a), tumor grades; (b), tumor stages; (c) and collectively (d).

N.S, nonsignificant correlation; S, Significant correlation; H.S, High significant correlation

**Figure 1:**
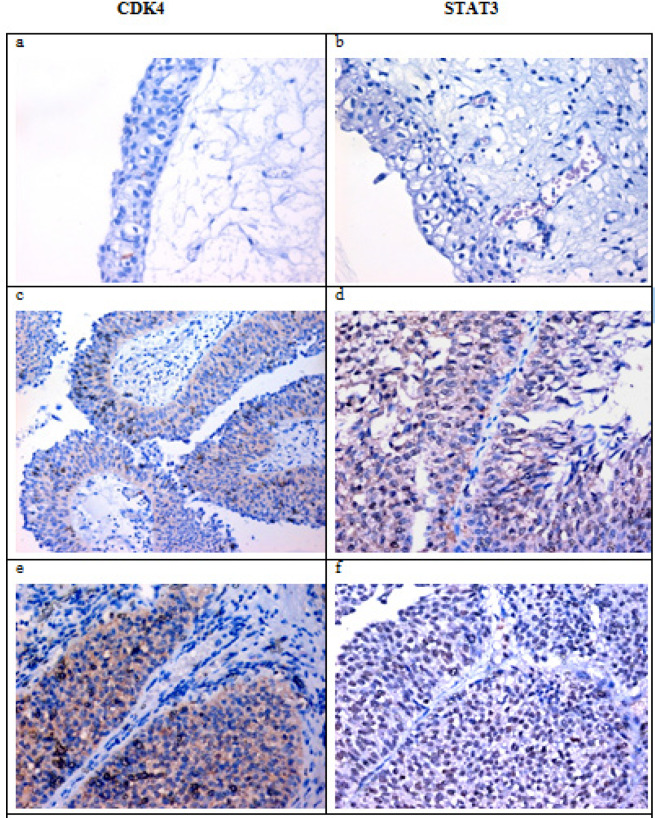
a and b showing normal urothelium with negative expression for both CDK4 and STAT4. c and d showed low grade papillary UC with low expression for CDK4(cytoplasmic) and STAT3(nuclear). e and f showed invasive papillary UCwith moderate expression of both markers. (IHC for CDK4 andSTAT3, DAB).

**Figure 2:**
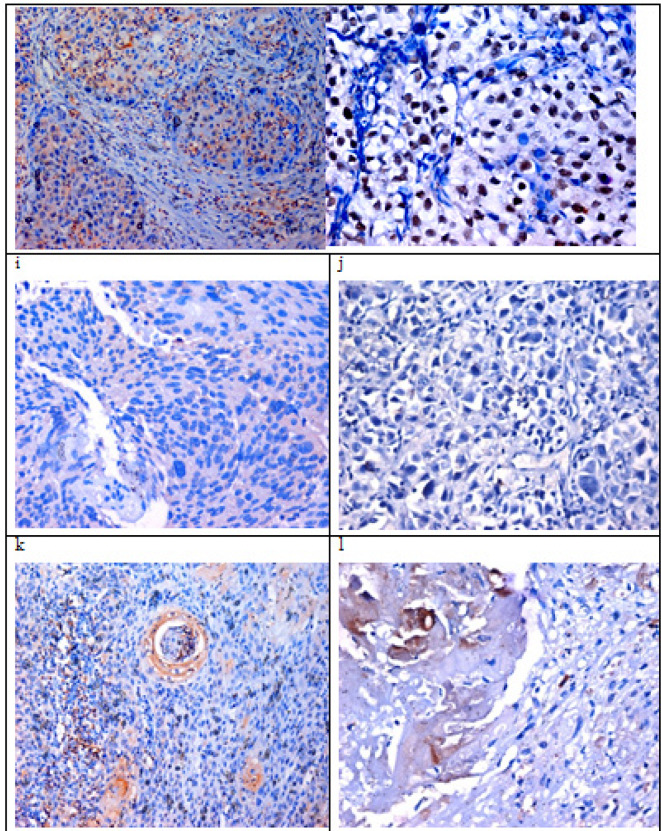
Invasive UC Showing Positive Cytoplasmic Expression for CDK4 and Marked Nuclear Expression for STAT3 (gandh respectively). iandj showed high grade Uc with mild expression of CDK4 and negative expression for STAT3. kandl showed a case of modetaely differentiated SCC with low expression of CDK4 and STAT3 in tumor cells (IHC for CDK4 andSTAT3, DAB).

## Discussion

Urinary bladder cancer (BC) is the second most common malignancy of all genitourinary tumors after prostate cancer with an incidence of 18.5 per 100,000 males and 5.7 per 100,000 females and approximately 25% of newly diagnosed bladder carcinoma patients present with aggressive muscle-invasive disease (Burger et al., 2013). In Egypt a significant decline in its frequency was observed becoming second after breast and contributing only 11 % of all cancers. Thus at the National Cancer Institute (NCI) (Moktar et al., 2007), pathology registry, bladder carcinoma constitutes 26 % of all cancers during time period (1985 – 1989), but dropped to only 12 % in years (2003 – 2004).The mean age was 46 years and male to female ration 4:1. This male predominance was most marked in squamous carcinoma (7:1) and least (2:1) in adenocarcinoma (El-Bolkainy et al., 2013). 

In the present study, according to the results shown there were no statistically significant differences in the percentage of diagnoses for male and females patients categories for both squamous cell carcinoma and urothelial carcinoma at 0.05 level. These results were in accordance with the results of (Fiorina et al., 2018) that showed no gender differences in the grade and the stages of either SCC or UC; however, the male-to-female ratio was higher for UC (5.9) than for SCC (2.7)

In our study, we found that 50% of squamous cell carcinoma and 66% of chronic cystitis cases were associated with bilharziasis but only 4.3% of the urothelial carcinoma patients were positive for bilharziasis. The difference between groups was statistically significant (p<0.001). 

Similar results were reported by (Liu et al., 2012) supporting our findings, as he stated that the percentage of patients (38.3%; 18 of 47) who had urothelial carcinoma with squamous differentiation was significantly higher than those with pure urothelial carcinoma (17.3%; 34 of 197; P <0 .01. Moreover, prior studies have reported that urothelial carcinoma with squamous differentiation is more aggressive because of its resistance to radiotherapy, chemotherapy, and immunotherapy (Gofrit et al., 2016).

According to (Felix et al., 2008), the main cause of SCC in developing countries is Schistosoma haematobium. Bladder cancer is one of the most prevalent malignancy among Egyptian males (16%), accounting for >7900 deaths per year, which is considerably higher than most other parts of the world (Feraly et al ., 2010 ).

Certainly, suitable molecular diagnostic markers are required to improve the early detection of bladder cancer and then to prolong survival of patients. The present study was aimed to explore the expression of *CDk4* and *STAT3 *in bladder cancer tissues.

*CDK4* is important for cell cycle G1 phase progression. The activity of this kinase is restricted to the G1-S phase, which is controlled by the regulatory subunits D-type cyclins and CDK inhibitor p16INK4a G1/S. Cyclin D-CDK4 complexes are major integrators of various mitogenic and antimitogenic signals. Also phosphorylates SMAD3 in a cell-cycle-dependent manner and represses its transcriptional activity. Component of the ternary complex, cyclin D/CDK4/CDKN1B, required for nuclear translocation and activity of the cyclin D-CDK4 complex. (Sheppard and McArthur, 2013). 

In our study, all cases of squamous cell carcinoma showed high tumor grade and positive muscle invasion, while most cases of urothelial carcinoma were of low grade malignancy (65.1%) and showed negative muscle invasion (70.0%). The difference was statistically significant (p<0.001). Another study that made on the non-bilharzial squamous cell carcinoma and transitional cell carcinoma, found that all squamous cell carcinoma patients were muscle invasive carcinoma patients and have staging at least pT2. This was in agreement with (Scosyrev et al., 2009) who stated that that squamous cell carcinoma have more aggressive than urothelial carcinoma in invasion and staging. On the other hand, they reported that in 205 patients with bilharzias bladder cancer; 122 (59.6%) were squamous cell carcinoma, 69 (33.7%) are urothelial carcinoma.

In our Immunohistochemical study, we show significant difference between groups of different bladder lesions considering the score and percentage of cellular expression of CDK4 (p< 0.01 and p <0.001 respectively). 

 Squamous cell carcinoma showed higher score of CDK4 expression compared to both chronic cystitis and urothelial carcinoma, with statistically significant difference (p<0.001 by ANOVA test).

Comparison between different studied groups showed higher values of CDK4 percent and score of expression in malignant tissues (SCC andUC) in relation to cystitis, with statistically significant differences (p<0.001 and p<0.01 respectively). However, significantly higher values of CDK4 percentage and scores of expression were detected in UC group compared to SCC group (p<0.01 and p<0.05 respectively).

Urothelial carcinomas with papillary patterns showed lower parameters of CDK4 expression compared to the non-papillary variant, with significant differences for CDK4 percent (p<0.01) and score (p<0.01).

Higher grades of UC showed significantly higher parameters of *CDK4* expression. This was true also if we add cases of squamous cell carcinoma to cases of urothelial carcinoma and make the scale of grade to be low and high collectively. 

Muscle invasion increases the level of *CDK4 *expression parameters, compared to non-muscle invasive UC. These differences were statistically significant for CDK4 score (p<0.05), while non-significant for CDK4 percent (p>0.1). 

Nucleo-cytoplasmic expression of CDK4 was found to be associated with higher levels of expression, compared to the cytoplasmic expression pattern, which was usually associated with lower levels of* CDK4* expression. The differences were statistically significant.

The relation between inflammation and cancer progression has been well established (Yu et al., 2009) has reported that STAT3 is the major inflammation-promoting transcription factor shown to play important roles in cancer progression in various types of tumors. Several studies have reported STAT3 as an important factor in the development of bladder cancer (Degoricija et al., 2014). 

In this study, we evaluated the relation between the expression of STAT3 pathway protein in the two most common types of bladder cancer; namely urothelial carcinoma and squamous cell carcinoma and the relation between *STAT3* expression and different grades and stages of bladder cancer, associated or non-associated with urinary bilharziasis in egyptian patients. 

The signal transducer and activator of transcription (STAT) proteins are intracellular transcription factors that mediate various aspects of cellular immunity, proliferation, apoptosis, and differentiation (Seif et al., 2017). The STAT family includes seven members (STAT1, STAT2, STAT3, STAT4, STAT5A, STAT5B, and STAT6). Among them, STAT3 has been shown to play a prominent role in tumor growth and invasion (Yu et al., 2009). In response to cytokines and growth factors, STAT3 is phosphorylated by receptor-associated Janus kinases (JAK), forms homo- or hetero-dimers, and translocates to the nucleus where it acts as a transcription activator (Yu et al., 2014).

Stat 3 is a latent transcription factor that normally resides in the cytoplasm. Upon growth factor/cytokine receptor or non-receptor tyrosine kinase-mediated activation, Stat3 rapidly translocates into the nucleus where it binds to consensus promoter region and activates target gene transcription (Ho et al., 2012).

Our findings comparing different studied groups showed higher values of STAT3 percent and score of expression in malignant tissues (SCC andUC) compared to cases of cystitis, with statistically significant differences (p<0.001 and p<0.01 respectively). however, significantly higher values of STAT3 percentage and scores of expression were detected in UC group compared to SCC group (p<0.01 and p<0.05 respectively). These results are in accordance with (Matsuzaki et al. 2018) univariate logistic regression analysis of variable parameters associated with patient prognosis. It showed that the STAT3 score, nuclear STAT3 score, pathological stage lymph node involvement, lymphovascular invasion, and tumor grade were associated with both progression-free survival and cancer-specific survival.

Two subtypes of bladder urothelial carcinomas exist: noninvasive papillary and muscle-invasive cancer. Evidence supports that these 2 subtypes develop through their own independent pathologic and molecular pathways, although certain overlap does exist (Goebell PJ., et al 2010) The vast majority of muscle-invasive cancers arise de novo from carcinoma in situ (CIS) without prior clinical progression through noninvasive papillary lesions (Wu, 2005) .

In our current study we have demonstrated that urothelial carcinomas with papillary patterns showed lower parameters of STAT3 expression compared to the non-papillary variant, with significant differences for STAT3 intensity (p<0.05), percent (p<0.01) and score (p<0.01). 

STAT3 has been implicated in the progression from carcinoma in situ to invasive bladder cancer . In particular, STAT3 signaling acts as an important downstream mediator of inflammatory cytokines, such as IL-6 and IL-17, which are released during bladder tumorigenesis due to chronic inflammation (i.e., smoking, persistent urinary tract infections) [Stat3 activation in urothelial stem cells leads to direct progression to invasive bladder cancer (Ho et al., 2012).

Present study shows the relation between STAT3 expression parameters and tumor grade of urothelial carcinoma (UC). Higher grades of UC showed significantly higher parameters of STAT3 expression. Comparable results has been reported by ( Matsuzaki et al., 2018), patients with high grade tumor , are patients with high STAT3 tumor and have a significantly higher risk of both disease progression (p = 0.008) and cancer-specific mortality (p = 0.008). 

Bladder cancer was proved by previous studies to be related to bilharzial infection, however, during our current work, we did not find statistically significant differences between bilharzial associated and non-associated bladder cancer. We suppose that the relation between STAT3 expression and bilharzial association was altered by the fact that most cases of bladder cancer that were associated with bilharziasis were of squamous carcinoma variant; which is almostly invasive in nature.

In conclusion, Our results showed a good correlation of the expression patterns of both the cell cycle (CDK4) and inflammatory (STAT3) markers studied and might be helpful for suggesting more selective agents in the therapeutic scenario of bladder cancer in the near future. Potential biomarkers such as CDK4 andSTAT3 may be targets for molecular based therapeutic strategies in the prevention or management of bladder cancer. Future studies should explore molecular mechanisms of proteins to define their roles in tumorigenesis.

## References

[B1] Amin, MB (2009). Histological variants of urothelial carcinoma: diagnostic, therapeutic and prognostic implications. Mod Pathol.

[B2] Black, PC, Brown, GA, Dinney CP (2009). The impact of variant histology on the outcome of bladder cancer treated with curative intent. Urol Oncol.

[B3] Burger M, Catto JW, Dalbagni G 20130. Epidemiology and risk factors of urothelial bladder cancer. Eur Urol.

[B4] Degoricija M, Situm M, Korać J (2014). High NF-κB and STAT3 activity in human urothelial carcinoma: a pilot study. World J Urol.

[B5] Dinney CP, McConkey DJ, Millikan RE (2004). Focus on bladder cancer. Cancer Cell.

[B6] Domanowska E, Jozwicki W, Domaniewski J (2007). Muscle-invasive urothelial cell carcinoma of the human bladder: multidirectional differentiation and ability to metastasize. Hum Pathol.

[B8] Erica JL, Weiguo Jn, Diana P, Keith L (2012). Stat3 activation in urothelial stem cells leads to direct progression to invasive bladder cancer philip levy Ho1 Syson chan Published in final edited form as. Cancer Res.

[B9] Felix AS, Soliman AS, Khaled H (2008). The changing patterns of bladder cancer in Egypt over the past 26 years. Cancer Causes Control.

[B10] Fiorina K, Christopher AL, Yun-Ling Z, George P, Sania A (2018). Urinary bladder cancer in Egypt: Are there gender differences in its histopathological presentation?. Adv Urol.

[B12] Goebell PJ, Knowles MA (2010). Bladder cancer or bladder cancers? Genetically distinct malignant conditions of the urothelium. Urol Oncol.

[B13] Gofrit ON, Yutkin V, Shapiro A (2016). The response of variant histology bladder cancer to intravesical Iimmunotherapy compared to conventional cancer. Front Oncol.

[B14] Ho PL, Kurtova A, Chan KS (2012). Normal and neoplastic urothelial stem cells: getting to the root of the problem. Nat Rev Urol.

[B15] Ho PL, Lay EJ, Jian W, Parra D, Chan KS (2012). Stat3 activation in urothelial stem cells leads to direct progression to invasive bladder cancer. Cancer Res.

[B16] Kim SP, Frank I, Cheville JC (2012). The impact of squamous and glandular differentiation on survival after radical cystectomy for urothelial carcinoma. J Urol.

[B17] Kujawski M, Kortylewski M, Lee H (2008). Stat3 mediates myeloid cell-dependent tumor angiogenesis in mice. J Clin Invest.

[B18] Liu C, Zhu Y, Lou W (2014). Inhibition of constitutively active Stat3 reverses enzalutamide resistance in LNCaP derivative prostate cancer cells. Prostate.

[B19] Liu X, He Z, Li CH (2012). Correlation analysis of JAK-STAT pathway components on prognosis of patients with prostate cancer. Pathol Oncol Res.

[B20] Matsuzaki K, Fujita K, Hayashi Y (2018). STAT3 expression is a prognostic marker in upper urinary tract urothelial carcinoma. PLoS One.

[B21] Mitra AP, Bartsch CC, Bartsch G Jr (2014). Does presence of squamous and glandular differentiation in urothelial carcinoma of the bladder at cystectomy portend poor prognosis? An intensive case-control analysis. Urol Oncol.

[B22] Mitra AP, Datar RHC, Malkowicz SB (2007). Muscle invasive urothelial carcinoma of the bladder. Urology.

[B23] Moktar N, Gouda I, El-Bolkainy T, El-Bolkainy MN (2007). Bilharziasis and badder cancer, a time trend analysisof 9843 patients. J Egypt NCI.

[B25] Scosyyrev EY, Messing E (2009). Urothelial carcinoma versus squamous cell carcinoma of bladder. Is survival different with stage adjustement. Urology.

[B26] Seif F, Khoshmirsafa M, Aazami H (2017). The role of JAK-STAT signaling pathway and its regulators in the fate of T helper cells. Cell Commun Signal.

[B27] Sheppard KE, McArthur GA (2013). The cell-cycle regulator CDK4: An emerging therapeutic target in Melanoma. Clin Cancer Res.

[B28] Stacey JB, Premkumar E (2013). CDK4: A key player in the cell cycle, development, and cancer. Reddy Genes and Cancer Reprints and permission.

[B29] Siegel R, Naishadham D, Jemal A (2013). Cancer statistics. CA Cancer J Clin.

[B30] Wasco MJ, Daignault S, Zhang Y (2007). Urothelial carcinoma with divergent histologic differentiation (mixed histologic features) predicts the presence of locally advanced bladder cancer when detected at transurethral resection. Urology.

[B31] Wu XR (2005). Urothelial tumorigenesis: a tale of divergent pathways. Nat Rev Cancer.

[B32] Yu H, Pardoll D, Jove R (2009). STAT3 in cancer inflammation and immunity: a leading role for STAT3. Nat Rev Cancer.

[B33] Yu H, Lee H, Herrmann A, Buettner R, Jove R (2014). Revisiting STAT3 signalling in cancer: new and unexpected biological functions. Nat Rev Cancer.

